# First Evidence of Sternal Wound Biofilm following Cardiac Surgery

**DOI:** 10.1371/journal.pone.0070360

**Published:** 2013-08-01

**Authors:** Haytham Elgharably, Ethan Mann, Hamdy Awad, Kasturi Ganesh, Piya Das Ghatak, Gayle Gordillo, Chittoor B. Sai-Sudhakar, Sashwati Roy, Daniel J. Wozniak, Chandan K. Sen

**Affiliations:** 1 Department of Surgery, The Ohio State University Wexner Medical Center, Columbus, Ohio, United States of America; 2 Davis Heart & Lung Research Institute, The Ohio State University Wexner Medical Center, Columbus, Ohio, United States of America; 3 Centers for Regenerative Medicine & Cell based Therapies, The Ohio State University Wexner Medical Center, Columbus, Ohio, United States of America; 4 Comprehensive Wound Center, The Ohio State University Wexner Medical Center, Columbus, Ohio, United States of America; 5 Department of Anesthesiology, The Ohio State University Wexner Medical Center, Columbus, Ohio, United States of America; 6 Department of Microbial Infection and Immunity and Microbiology, The Ohio State University Wexner Medical Center, Columbus, Ohio, United States of America; 7 Department of Plastic Surgery, The Ohio State University Wexner Medical Center, Columbus, Ohio, United States of America; Columbia University, United States of America

## Abstract

Management of deep sternal wound infection (SWI), a serious complication after cardiac surgery with high morbidity and mortality incidence, requires invasive procedures such as, debridement with primary closure or myocutaneous flap reconstruction along with use of broad spectrum antibiotics. The purpose of this clinical series is to investigate the presence of biofilm in patients with deep SWI. A biofilm is a complex microbial community in which bacteria attach to a biological or non-biological surface and are embedded in a self-produced extracellular polymeric substance. Biofilm related infections represent a major clinical challenge due to their resistance to both host immune defenses and standard antimicrobial therapies. Candidates for this clinical series were patients scheduled for a debridement procedure of an infected sternal wound after a cardiac surgery. Six patients with SWI were recruited in the study. All cases had marked dehiscence of all layers of the wound down to the sternum with no signs of healing after receiving broad spectrum antibiotics post-surgery. After consenting patients, tissue and/or extracted stainless steel wires were collected during the debridement procedure. Debrided tissues examined by Gram stain showed large aggregations of Gram positive cocci. Immuno-fluorescent staining of the debrided tissues using a specific antibody against staphylococci demonstrated the presence of thick clumps of staphylococci colonizing the wound bed. Evaluation of tissue samples with scanning electron microscope (SEM) imaging showed three-dimensional aggregates of these cocci attached to the wound surface. More interestingly, SEM imaging of the extracted wires showed attachment of cocci aggregations to the wire metal surface. These observations along with the clinical presentation of the patients provide the first evidence that supports the presence of biofilm in such cases. Clinical introduction of the biofilm infection concept in deep SWI may advance the current management strategies from standard antimicrobial therapy to anti-biofilm strategy.

## Introduction

Median sternotomy is the most common approach used in cardiac surgery procedures to access the heart. The incidence of human sternotomy wound site infection ranges from 1–8% [1], [2], [3]. This incidence is significant, both economically as well as with respect to health care impact, considering the annual volume of patients undergoing cardiac surgery procedures (more than 600,000 cardiac surgeries per year) and associated health complications [4]. Sternal wound infection (SWI) is a serious complication even after successful surgery with mortality rates reaching 40% [2], [5], [6]. Additionally, patients with SWI require prolonged antibiotic courses, repeated surgical interventions, longer hospital stay, and eventually higher health care cost [2]. One of the challenges in the management of these patients is overt clinical signs of infection despite ambiguous or negative culture results.

One of the most common pathogens isolated from SWI are Gram positive bacteria, with Staphylococci species being the most frequently reported [7], [8], [9]. Staphylococci strains (such as *S. aureus* & *S. epidermidis*) are known for their ability to grow as biofilms [10]. Once a component of the biofilm structure, the bacterial cells become recalcitrant to the host immune responses as well as antimicrobial therapies, resulting in persistent infection [11]. Microorganisms attach to the surfaces such as wound tissues, biomaterials and surgical implants (*e.g.* sutures and hard ware-stainless steel wires) to form biofilms [12], [13], [14]. Colonization of surgical wounds with biofilm makes them resistant to both antimicrobial as well as other interventions such as surgical debridement aimed at treating wound infection [14], [15]. Given the poor prognosis of cardiac surgery wound infection complications, we sought to look for the presence of biofilm at the sternal wound site in patients undergoing cardiac surgery. This work provides the first direct evidence demonstrating presence of biofilm infection in sternal wound site cardiac surgery patients. The introduction of the concept of biofilm infection in deep SWI will help revisit wound management strategies.

## Results

**Figure 1:**
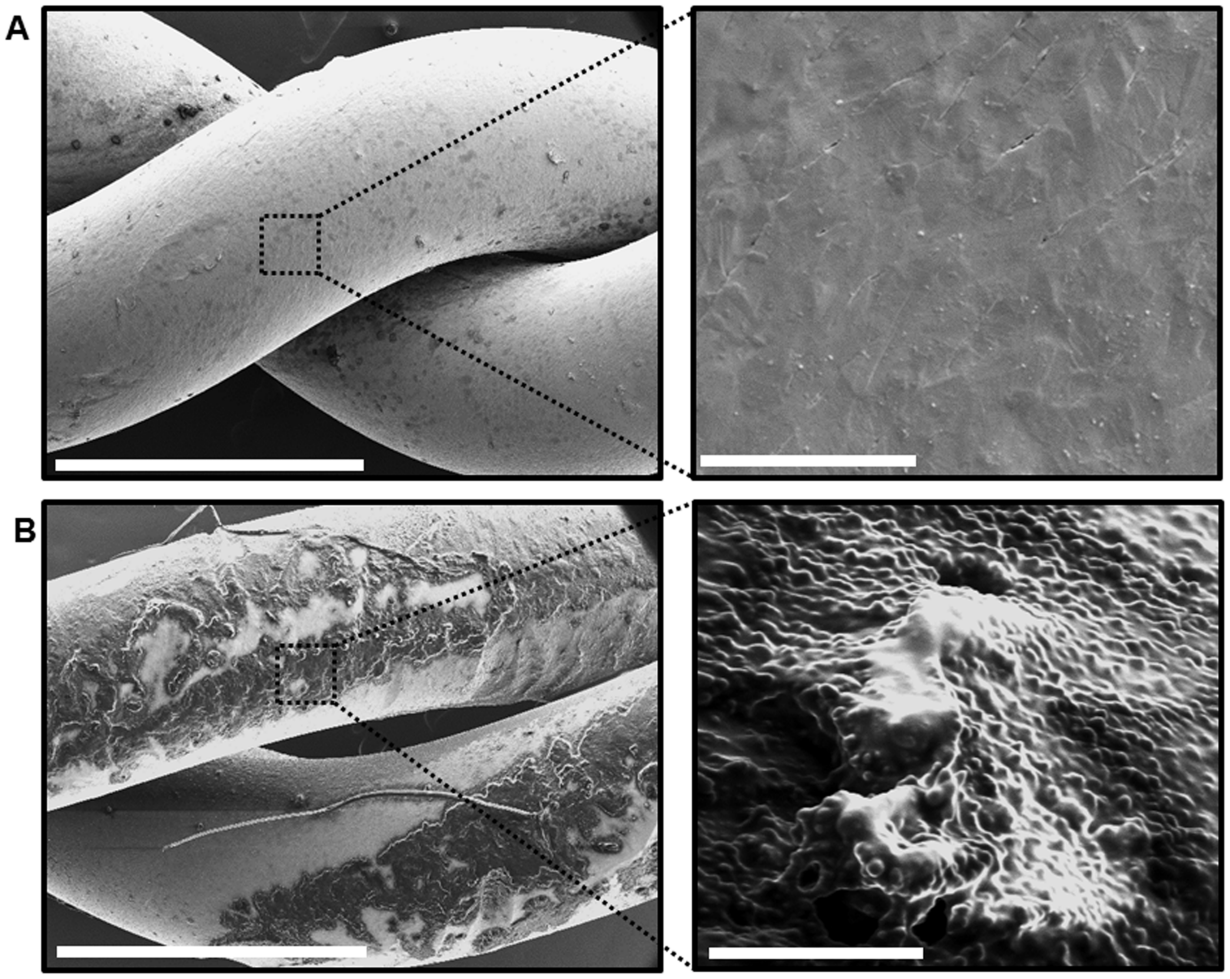
Scanning electron microscopy images of infected stainless steel wires used for sternotomy closure. (A) Left panel is a SEM at 60x magnification of an unused sterile stainless steel wire, twisted in a way similar to that after sternotomy closure. Scale bar = 1 mm. Right panel is a higher magnification (10,000x) of the dashed box area in the left panel, showing the metal surface of the wire. Scale bar = 5 µm (B) Left panel is a SEM at 60x magnification of stainless steel wire after overnight incubation with MRSA strain USA300. Scale bar = 1 mm. Note the wire metal surface is coated by a film of material. Right panel is a higher magnification (10,000x) of the dashed box area in the left panel, showing clusters of cocci attached to the extracted wire and embedded within amorphous slime. Scale bar = 5 µm.

Stainless steel wires used for approximation of the sternum after cardiac surgery were tested *in vitro* for bacterial adhesion, biofilm formation, and recalcitrance to antimicrobial tobramycin. In the SWI cultures from patients, both Methicillin-resistant Staphylococcus aureus (MRSA) and Methicillin-sensitive Staphylococcus aureus (MSSA) were identified (Table 1). Methicillin resistance is independently associated with increased mortality and hospital charges among patients with *S. aureus* surgical site infections (SSI), therefore, we chose MRSA for in vitro studies [17]. Wires were twisted in a manner similar to that done during closing of sternotomy in the operating room and then incubated with MRSA PFGE strain type USA300 (source, Los Angeles correctional facility), for 24 h. Other wires from the same stock were used as un-inoculated controls. Examination of the wires under scanning electron microscope (SEM) showed attachment and accumulation of MRSA isolates on the wires within extracellular amorphous material forming three-dimensional structures (Fig. 1B). SEM imaging of the control wires showed no microorganisms attached to the metal surface (Fig. 1A).

**Table 1:** Demographic characteristics of patients (n = 9) and SWI status.

Subjects	SWI	Age	Sex	BMI	Associated medical conditions	Procedure	Antimicrobial therapy	Time interval between procedure and debridement	Woundculture	Bloodculture	Data shown in Figure #
**SW001**	**Yes**	60	M	34.7	CAD-DM-HTN-HLD-RF	CABG	Nafcillin, Daptomycin	5 weeks	MSSA	N	3A, 4,6
**SW002**	**Yes**	84	F	40.9	CAD-HTN-HLD-RD-COPD	Redo-MVR	Ertapenem	2 weeks	negative	N	6
**SW003**	**Yes**	61	F	18	CAD-HTN-HLD-PVD	LVAD	Vancomycin, Ciprofloxacin, Sulfamethoxazol, e-triethoprim, Linezoid	12.1 weeks	No growth	N	7
**SW004**	**Yes**	54	M	27	DM-HTN-RD-OSA	CABG	Piperacillin-tazobactam, Vanomycin	3.1 weeks	MRSA	N	3B & 5
**SW005**	**Yes**	64	M	25.1	CAD- COPD-HLD-DM	PM-Repair of RV	Piperacillin-tazobactam, Vancomycin, Daptomycin	5.4 weeks	No growth	N	3A
**SW006**	**No**	28	M	23.7	END-SEP	excision scar					Not shown
**SW007**	**Yes**	43	F	41	HTN P HTN-RHD	MVR	Linezolid	9.2 weeks	No growth	MRSA	6
**SW008**	**No**	46	F	50.2	HTN P-OSA-GERD-AKI	LRB					7
**SW009**	**No**	31	M	20.7	CGH-SVT	AVR					5

M, male, F, female; AKI, acute kidney disease; BMI, body mass index, CAD, coronary artery disease; CGH, coronary heart disease; DM, diabetes mellitus; END, endocarditis; GERD, gastro esophageal reflux disease; HTN, hypertension; HTN- P, Pulmonary hypertension; HLD, hyperlipidemia; RD, renal dysfunction; COPD, chronic obstructive pulmonary disease; PVD, peripheral vascular disease; OSA, obstructive sleep apnea; RHD, rheumatic heart disease; CABG, coronary artery bypass graft; MVR, mitral valve replacement; LVAD, left ventricular assisted device; PM, pace maker; RV, right ventricle; N, negative; MSSA, Methicillin-sensitive *Staphylococcus aureus*; MRSA, Methicillin-resistant *Staphylococcus aureus;* SVT, supraventricular tachycardia.

**Figure 2:**
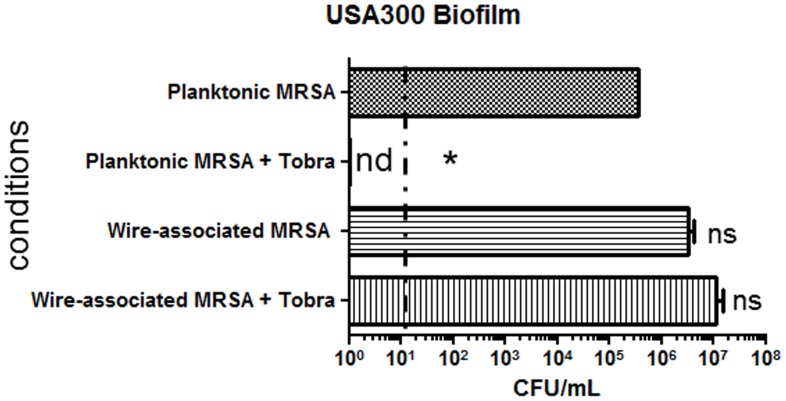
MRSA Strain USA300 biofilm exhibits enhanced tolerance to tobramycin when grown as a biofilm on surgical wires. USA300 was used to inoculate in vitro wells containing sections of wire. Planktonic bacteria and wire-associated biofilms were challenged with 10 ug/ml of tobramycin for 2 hours. Bacteria tolerant to antibiotic challenge were enumerated using viability plating and compared to untreated parallel controls. Percent survivability of triplicate cultures is represented. nd, not detected, ns, not significant. Data are mean±SD (n = 3), *p<0.05 compared to untreated planktonic (Mann Whitney test).

Biofilms associated with biomedical implant infections are known for their resistance to antibiotics [18]. To determine whether wire-associated bacteria show characteristics of classical biofilm bacteria described in the literature, the wires were inoculated with MRSA and challenged with tobramycin. The resistance to tobramycin was evaluated in wire-associated bacteria versus planktonic bacteria. After 2-h of challenge, tobramycin failed to kill wire-associated bacteria but reduced the planktonic load greater than five-log (Fig. 2).

**Figure 3:**
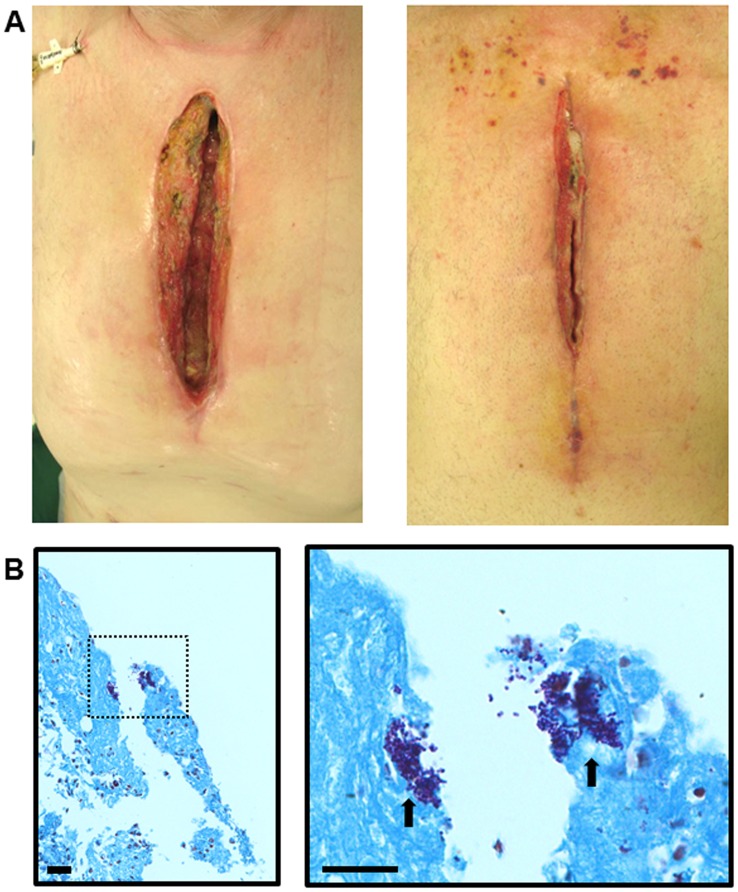
Digital photos and gram staining of deep sternal wound infection in two patients scheduled for a debridement procedure. (A) Digital photos of the infected sternal wounds. Note the signs of active infection with localized erythema, exudates, friable wound edges and sternal instability. Sternal wires were removed before the debridement procedure. (B) Gram-Twort staining of debrided tissues taken of infected sternal wound showing clumps of Gram-positive cocci (arrows in right panel). Left panel, scale bar = 50 µm, 400x magnification. Right panel is the zoom of the dashed boxed area in the left panel (scale bar = 50 µm).

For the clinical study, nine patients were recruited. Three of the nine patients (control non SWI) had a cardiac surgery procedure previously and were scheduled for a second surgical procedure in which they underwent re-sternotomy. The sternotomy wound sites in the three patients were intact with an old scar and no signs of infection were noted. In the test arm, remaining six patients had deep sternal wound infection (SWI) which complicated their cardiac surgery and were therefore scheduled for a sternal debridement procedure (SWI group). These wounds were initially classified as infected by the physician providing care using standard clinical criteria including systemic leukocytosis/fever and localized signs of infection including erythema, necrosis, discharge, and failure of healing. The infection involved the skin, subcutaneous tissue, and extended to the sternum. The sternotomy wound site displayed signs of active infection with localized erythema, exudates, friable wound edges and sternal instability (Fig. 3A). The average interval between the cardiac surgery procedure and the debridement procedure was 2 to 12 weeks in which different classes of antibiotics were used to manage infection (Table 1). Wound cultures showed colonization with MSSA, MRSA in two and other four showed negative culture data.

As an initial screening method, the debrided tissues taken from infected sternal wounds were stained using Gram staining. The staining showed patchy pattern of colonization with numerous Gram positive cocci. Some areas of the tissues showed extensive colonization with large aggregates, while other areas were infected with small micro-colonies or single cocci (Fig. 3B).

**Figure 4:**
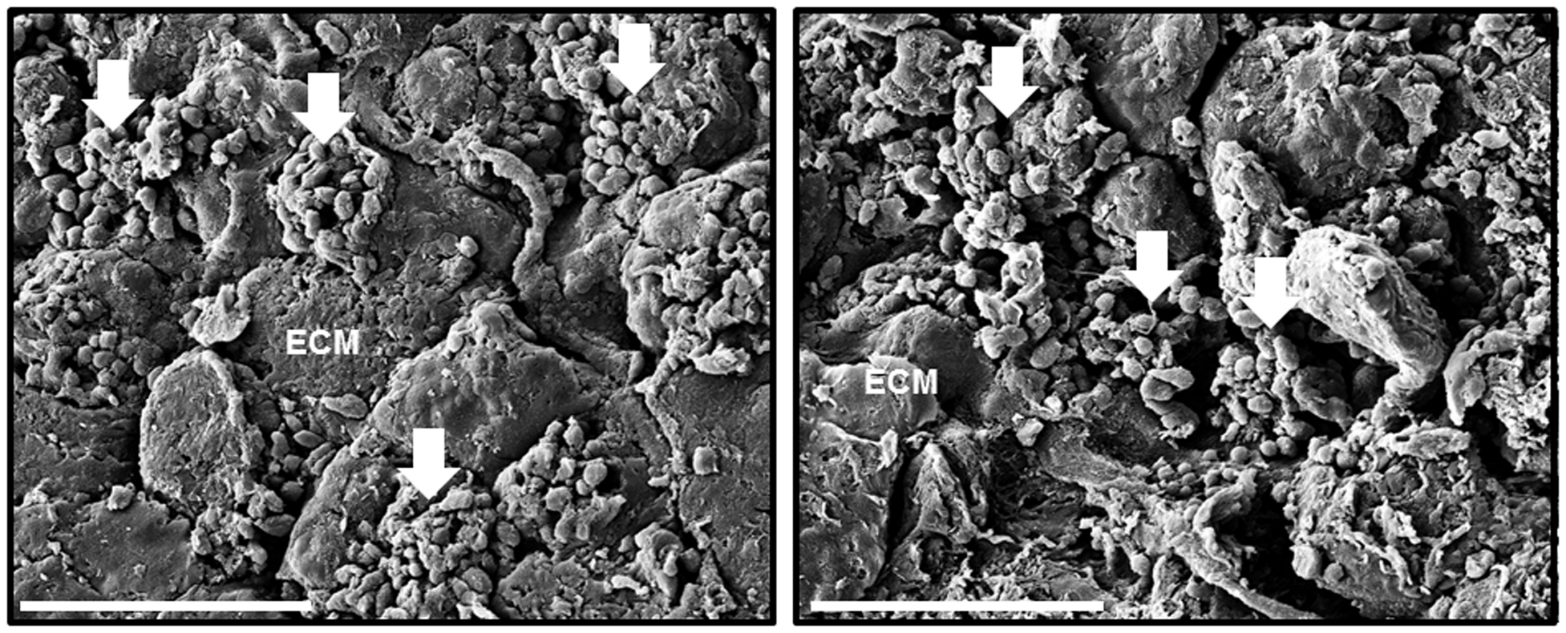
Scanning electron microscopy images of debrided tissues taken from infected sternal wound. Representative scanning electron microscopy images showing clusters of cocci (arrows) attached to the tissues. ECM, extra-cellular matrix. Scale bar = 10 µm, 5000x magnification.

To visualize bacterial aggregates in the wound tissue, the surfaces of debrided tissue samples were analyzed using SEM imaging. The samples showed scattered aggregates of cocci attached to the wound surface and appeared as three-dimensional structures (Fig. 4). The bacteria were partially covered with extracellular fibers connecting the cocci together within the three-dimensional structure.

**Figure 5:**
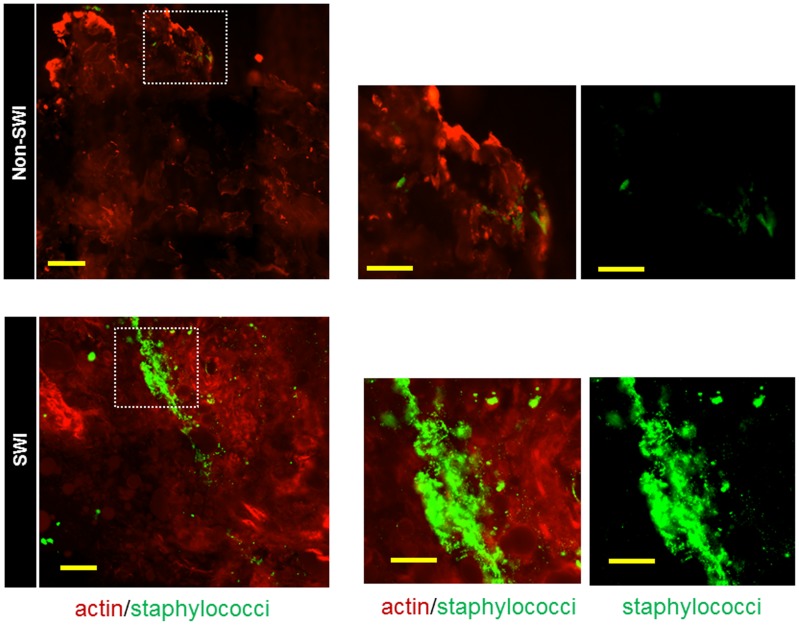
Presence of staphylococci within the infected debrided wound tissues. Representative confocal microscopy images of debrided tissue using immunofluorescence staining (debrided tissues was counterstained red with Phalloidin). Note large aggregates of staphylococci (intense green granular stain) colonizing the debrided tissues of infected sternal wound (lower panels), while tissues taken from a non-infected sternal wound during resternotomy (upper panels) show no colonization with staphylococci. Scale bar = 50 µm, 400x magnification. (SWI: sternal wound infection). Right panel is the zoom of the dashed boxed area in the left panel (scale bar = 20 µm).

According to previous clinical reports, staphylococcus is one of the most common organisms isolated from patients with SWI [7], [8], [9]. The culture reports from two out of six SWI patients showed MRSA colonization at wound site (Table 1). Morphological analysis of Gram stain and SEM images also suggested staphylococci infection. Identification of staphylococci in the debrided tissues was confirmed by immuno-fluorescence staining using anti-staphylococci antiserum. Indeed, tissue samples taken from infected sternal wounds were showed discrete intense granular green stain indicative of micro colonies of staphylococci (Fig. 5, *lower panels,*). No evidence of staphylococci was found in tissues taken from non-infected re-sternotomy wound of patients in the control arm (Fig. 5, *upper panels*).

**Figure 6:**
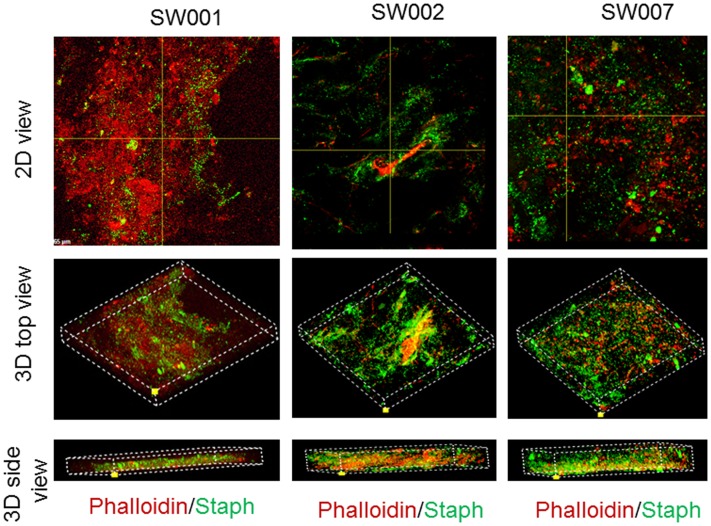
Confocal laser scanning microscopy images showing three dimensional presence of staphylococci in infected debrided wound tissue. Z-stack image created by merging serial scans of thick tissue section (20 µm), viewed under 600x magnification confocal laser scanning microscopy. Showing in the *x/y* plane clumps of staphylococci colonizing the debrided tissues (red), while the *x/z* and *y/z* planes display the depth of the colonization throughout the tissue section. Three-dimensional orthogonal projections of z-stack image in panel (oriented in two different planes) showing of staphylococci aggregates biomass within the debrided tissues.

The architecture of staphylococci micro colonies within the debrided tissues was further studied using confocal laser scanning microscope (CLSM) (Fig. 6). Three-dimensional images were developed to visualize the depth of staphylococci biofilms throughout thick tissue sections (20 µm). Most of the staphylococci were organized in three-dimensional clumps that were scattered across tissue sections (Fig. 6). Together, these clumps constituted a thick staphylococci biomass that traversed through over 70% of the whole tissue section (Fig. 6).

**Figure 7:**
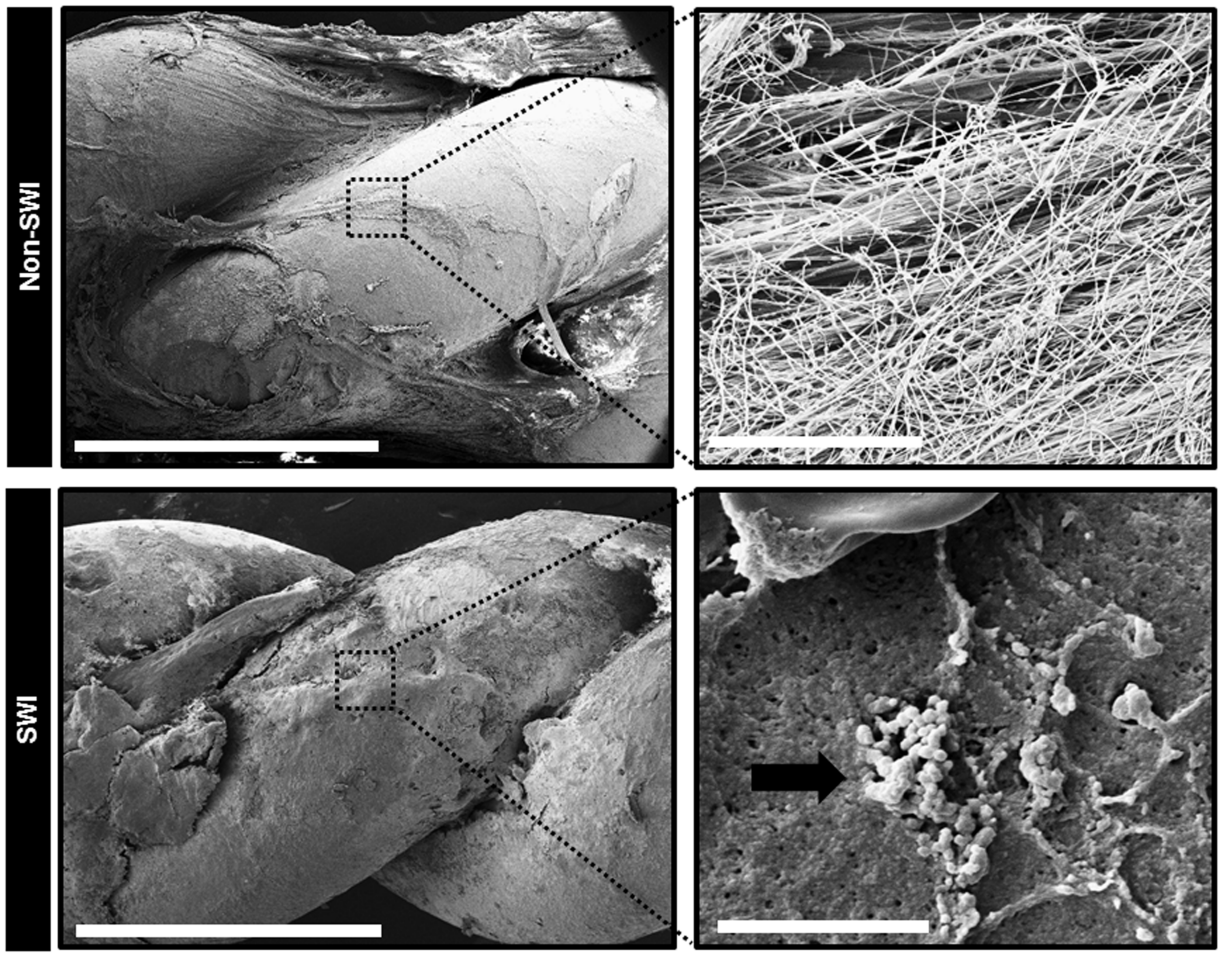
Scanning electron microscopy images of extracted stainless steel wire taken from infected/non-infected sternal wounds during a re-sternotomy procedure. Upper panels, left. Scanning electron microscopy (SEM) image at 60x magnification of extracted stainless steel wire taken from a non-infected sternal wound during a re-sternotomy procedure. Scale bar = 1 mm. Right panel is a higher magnification (10,000X) of the dashed box area in the left panel, no aggregates of cocci were found. Scale bar = 5 µm. Lower panel left. SEM image at 60x magnification of extracted stainless steel wire taken from infected sternal wound during a debridement procedure. Scale bar = 1 mm. Right panel is a higher magnification (10,000x) of the dashed box area in the left panel, showing three-dimensional aggregate of cocci (black arrow) attached to the extracted wire. Scale bar = 5 µm. (SWI, sternal wound infection).

Stainless steel wires extracted from infected or non-infected sternal wound were examined under SEM. The metal surface of the wires was completely coated by mix of extracellular tissue matrix, fibers, and red blood cells. Interestingly, in test patients, we observed three-dimensional clusters of cocci attached to the hardware extracted from infected sternal wound (Fig. 7, *lower panels*). Such clusters of cocci were not found in wires from non-infected sternal wound of control patients (Fig. 7, *upper panels*). Additionally, we note that staphylococci were never isolated from non-infected sternal wound hardware using standard culture methods *(data not shown).*

## Discussion

Post-sternotomy wound infections are classified into superficial and deep. Superficial sternal wound infections (SWI) are confined to the skin and/or subcutaneous tissue with overall good response to antimicrobial therapies and local wound care. On the other hand, deep SWI includes, besides the superficial wound infection, sternal osteomyelitis with or without infection of the retrosternal structure [2]. Despite being uncommon, deep SWI is a life threatening complication after cardiac surgery with associated high mortality (10 - 40%) [2], [5], [6]. Antimicrobial therapies alone usually fail to successfully treat deep SWI, which necessitates adding physical therapies such as surgical debridement, vacuum-assisted closure, rigid sternal fixation, and flap reconstruction [19]. These interventions are highly limited in productivity as the incidence of mortality in these case remains high [20], [21]. In particular, non-responsiveness of post-sternotomy deep wound infections to broad spectrum antibiotics remains a major clinical challenge [19], [22].

In the majority of SWI clinical reports, microbiological analysis revealed wound colonization with staphylococcal strains (methicillin-sensitive *Staphylococcus aureus,* methicillin-resistant *Staphylococcus aureus, Staphylococcus epidermidis)* [7], [8], [9], [23], [24]. Staphylococcus strains are known for their capability to form robust biofilms on exposed tissues or biomaterials surfaces [10], [25]. Staphylococcus species are the most important pathogen responsible for biofilm-associated medical devices infection [18], [26], [27]. Staphylococci biofilms have been identified on intravascular catheters, endocardial pacemaker lead, vascular grafts, mechanical heart valves, orthopedic implants, and ventriculo-peritoneal shunts [28], [29], [30], [31], [32], [33]. We noted that MRSA clinical isolates were able to accumulate on the wires and grow as three dimensional aggregates of cocci encased in an amorphous extracellular material. These MRSA aggregates on the wires displayed resistance to tobramycin compared to planktonic isolates. These data are consistent with previous report where Olsson et al. compared the adherence ability of staphylococcal clinical isolates to sternal fixation stainless steel wires *in vitro* [34]. They reported no difference in adherence and attachment between coagulase negative staphylococci isolated from deep SWI and contaminants of non-infected re-sternotomy wounds. However, accumulation as biofilms on the wires were more frequently observed in deep SWI isolates than in contaminants [34].

Following clinical diagnostic criteria of biofilm associated infections as proposed by Parsek and Singh were evaluated in this study: (a) Infecting bacteria were adherent to some substratum or are surface associated; (b) direct examination of infected tissue showed bacteria living in cell clusters, or micro colonies, encased in extracellular matrix; (c) the infection confined to a particular location although secondary dissemination is possible and (d) antibiotic resistance despite the fact that the responsible organisms are susceptible to killing in the planktonic state [35]. Scanning electron microscopy detected three-dimensional aggregates of cocci attached to the wound tissues and stainless steel wires. Confocal laser scanning microscopy helped visualize thick layers of three-dimensional staphylococci aggregates distributed throughout the debrided tissue sections. The infection was localized to the sternotomy wound site with negative blood cultures and no signs of systemic involvement except in one patient. All patients were scheduled for surgical debridement after unsuccessful extended rounds of different classes of broad spectrum antibiotics to control the infection. Matching these clinical findings and the *in vitro* work with the clinical diagnostic criteria mentioned above, we conclude that deep SWI in the six patients of the test arm is a biofilm-associated infection.

Biofilm infected-wounds represent a real clinical challenge because of the complex nature, in which different bacterial species act synergistically to survive together [36], [37], [38]. Not only are these populations resistant to the host immune response and antibiotics, but also many bacteria inside biofilms are difficult to be identified by standard culture methods [39], [40]. This may explain why only 2 out of 6 subjects had positive cultures report using standard clinical diagnostics, while staphylococci biofilm was detected by histological analysis in all of these patients. This observation indicates a reason for the failure of standard antimicrobial therapies to control the infection suggesting a comprehensive biofilm directed strategy is required to manage such infections. Although debridement decreases the bacterial burden, removes devitalized tissues (nutrient sources for bacteria), and enhances the immune response (by improving microcirculation), it fails to completely eradicate all pocket-containing bacteria within the wound [41], [42]. Concerns have been raised regarding the efficacy of debridement alone against wound-associated biofilm infection [43]. Debridement also increases the wound size attenuating previous re-epithelialization and wound closure progression. Bacterial colonies, missed by debridement, can invade the wound bed again, reattach, and establish biofilm communities within 48 hours post-debridement [44]. Current medical treatment of SWI includes empiric antibiotic therapy with broad coverage that will be changed to specific therapy according to cultures [19]. There is almost a need of surgical interference, along with the medical therapy, to overcome the infection. According to our findings in the current study, the resistant nature of SWI to medical therapy is attributed to biofilm-associated infection. We suggest that combined therapies are needed to improve the outcomes. Debridement to be applied early to reduce the microbial load, removes necrotic tissues and foreign bodies, and physically disperses complex bacterial communities within the wound. This will force bacterial cells to enter a growing phase and actively search for a new site for reattachment. During which, a combination of anti-biofilm agents and newly developed antibiotics such as Daptomycin or Linezolid should be administrated [22], [45], [46]. Molecular analysis of the wound microbiology is needed to target other uncultivable pathogens might be involved in deep SWI. Additionally, coating of sternal fixation wires with anti-biofilm agents should be considered for further research. Introduction of anti-biofilm agents as part of wound care was shown to enhance the healing of chronic wounds in a recent clinical study [47]. One of the anti-biofilm agents, ribonucleic-acid-III–inhibiting peptide (RIP), has received significant attention regarding staphylococcus infections [48]. RIP is a regulator molecule of quorum sensing in staphylococcus that was shown to suppress genes responsible for toxins production and biofilm formation in experimental infection models [48].

In summary, this work provided the first direct evidence demonstrating biofilm in deep SWI. Introduction of biofilm concept in deep SWI will help revisit management strategies. New classes of drugs have been developed to target different steps of biofilm formation: attachment, extracellular matrix, cell-to-cell communication (quorum sensing), and biofilm dispersion [49], [50]. Consideration of such anti-biofilm strategies to manage deep surgical wound infection in cardiac surgery patients is warranted.

## Materials and Methods

### In vitro Biofilm Culture on Surgical Steel Wires

USA300 MRSA cultures [16] were grown in tryptic soy broth (TSB) for 18 h to prepare an inoculum. The 18 h culture was diluted in fresh TSB to an optical density (OD_600_) equal to 0.1 and inserted into replicate wells of a 12-well plate. Sterile wires commonly used for sternal fixation after cardiac surgery were twisted and cut into 15 mm sections and placed into each of the wells containing the USA300 inoculum. The plates were incubated at 37°C and 5% CO_2_ on an incubator for 24 h. Following the growth period, planktonic cells were pelleted at 20,000xg and washed using PBS while wires were dipped into fresh PBS for washing. Planktonic cells and wire-associated cells were treated with 10 µg/ml tobramycin for 2 h. Following treatment, cells were sonicated for 10 minutes in a bath sonicator. The suspensions were serially diluted and plated. Tobramycin untreated planktonic or wire-associated cells were used as controls and each case the untreated colony forming units (CFU) were approximately 10^6^ CFU/ml. Percent survival was calculated using log (treated CFU)/log (untreated CFU)x100.

### Human Subjects and Sample Collection

Subjects (*n* = 6) participating in the study were patients scheduled for debridement procedure of an infected sternal wound after cardiac surgery or a re-sternotomy procedure for a second cardiac surgery with healed non-infected sternal wound (control, *n* = 3). All procedures were done at the Wexner Medical Center of The Ohio State University. All approvals were obtained as required. Our study protocol was approved by the Biomedical Sciences Institutional Review Board at The Ohio State University and all participants provided informed written consent. Demographic characteristics of patients and wound related information are listed in Table 1. Protocol was approved by the Ohio State University’s Institutional Review Board. Samples were obtained from individual subjects during the debridement or re-sternotomy procedure. The upper part of the debrided tissue, representing the surface of the wound bed, was kept in a fixative solution for imaging by SEM. The rest of the tissue sample was kept in 4% formalin or immediately embedded in optimum cutting temperature (OCT) compound and stored frozen in liquid N_2_ for histological analyses. Stainless steel wires, commonly used for closing of sternum, were collected in a fixative solution for imaging by SEM.

### Wound Culture Clinical Procedures

Wound cultures were done in Ohio State University Wexner Medical Center Clinical microbiology laboratory using standard culture procedures. In brief, wound specimens were plated on selective media for culture and examinations. The identification and susceptibility testing on following was performed: i) gram negative rod; ii) s. aureus; iii) iii) enterococcus.

### Histology

Formalin-fixed, paraffin-embedded or OCT-embedded frozen wound-edge specimens were sectioned. The paraffin sections (5 µm) were de-paraffinized and stained with Gram/Twort stain (Newcomer Supply Inc., Middleton, WI) using standard procedures. Immunofluorescence staining of frozen sections (8 µm) was performed using anti-staphylococci antibody (1∶500; Abcam®, Cambridge, MA; ab20920) and Alexa Fluor® 568 Phalloidin (1∶200; Life Technologies™, Grand Island, NY). Fluorescence detection and counterstaining were performed by using Alexa Fluor® 488 secondary antibody (1∶200, Life Technologies™, Grand Island, NY).

### Imaging

Fluorescence stained sections were imaged using a Zeiss Axiovert 200 inverted fluorescent microscope supported by an AxioCam digital camera, a motorized stage and guided by Axiovision software (Zeiss, Thornwood, NY). Mosaic images of Gram/Twort or immunofluorescence stained debrided tissues were collected.

### Confocal Scanning Laser Microscope Imaging

The biomass of staphylococci clumps colonizing the debrided tissues was visualized using an Olympus Fluoview FV1000 spectral confocal microscope (Olympus, Pittsburgh, PA) under 600X magnification using an argon laser. Z-stack images were created by merging serial scans of thick tissue section (20 µm). Three-dimensional orthogonal projections of the z-stack images were generated by the Fluoview FV1000 software.

### Scanning Electron Microscope (SEM) Imaging

Debrided tissue samples and stainless steel wires were fixed in a 2.5% glutaraldehyde solution in 0.2 M phosphate buffer for 3 days. On the 4th day, samples were washed with 0.1 M phosphate buffer and then dehydrated using graded concentrations of ethanol. Samples were washed with hexamethyldisilazane (HMDS, Ted Pella Inc., Redding, CA) and left to dry overnight. Before scanning, samples were mounted and coated with gold. A FEI™ NOVA nano SEM (FEI™, Hillsboro, OR) equipped with a field-emission gun electron source was used for imaging.

## References

[1] Mauermann WJ, Sampathkumar P, Thompson RL (2008) Sternal wound infections. Best Pract Res Clin Anaesthesiol 22: 423–436. 18831296 10.1016/j.bpa.2008.04.003

[2] El Oakley RM, Wright JE (1996) Postoperative mediastinitis: classification and management. Ann Thorac Surg 61: 1030–1036. 8619682 10.1016/0003-4975(95)01035-1

[3] Negri A, Manfredi J, Terrini A, Rodella G, Bisleri G, et al (2002) Prospective evaluation of a new sternal closure method with thermoreactive clips. Eur J Cardiothorac Surg 22: 571–575. 12297174 10.1016/s1010-7940(02)00411-6

[4] Owens S, Ramraj V, Wallop J (2010) The Cardiac Surgery Advanced Practice Group: A Case Study of APN and PA Collaborative Practice. The journal for nurse practitioners 6: 371–374.

[5] Tavilla G, van Son JA, Verhagen AF, Lacquet LK (1991) Modified Robicsek technique for complicated sternal closure. Ann Thorac Surg 52: 1179–1180. 1953149 10.1016/0003-4975(91)91310-r

[6] Gummert JF, Barten MJ, Hans C, Kluge M, Doll N, et al (2002) Mediastinitis and cardiac surgery–an updated risk factor analysis in 10,373 consecutive adult patients. Thorac Cardiovasc Surg 50: 87–91. 11981708 10.1055/s-2002-26691

[7] Poncelet AJ, Lengele B, Delaere B, Zech F, Glineur D, et al (2008) Algorithm for primary closure in sternal wound infection: a single institution 10-year experience. Eur J Cardiothorac Surg 33: 232–238. 18082415 10.1016/j.ejcts.2007.11.016

[8] Sharma M, Berriel-Cass D, Baran J Jr (2004) Sternal surgical-site infection following coronary artery bypass graft: prevalence, microbiology, and complications during a 42-month period. Infect Control Hosp Epidemiol 25: 468–471. 15242193 10.1086/502423

[9] Stahle E, Tammelin A, Bergstrom R, Hambreus A, Nystrom SO, et al (1997) Sternal wound complications–incidence, microbiology and risk factors. Eur J Cardiothorac Surg 11: 1146–1153. 9237601 10.1016/s1010-7940(97)01210-4

[10] Otto M (2008) Staphylococcal biofilms. Curr Top Microbiol Immunol 322: 207–228. 18453278 10.1007/978-3-540-75418-3_10 PMC2777538

[11] Costerton JW, Stewart PS, Greenberg EP (1999) Bacterial biofilms: a common cause of persistent infections. Science 284: 1318–1322. 10334980 10.1126/science.284.5418.1318

[12] Costerton JW, Montanaro L, Arciola CR (2005) Biofilm in implant infections: its production and regulation. Int J Artif Organs 28: 1062–1068. 16353112 10.1177/039139880502801103

[13] Lynch AS, Robertson GT (2008) Bacterial and fungal biofilm infections. Annu Rev Med 59: 415–428. 17937586 10.1146/annurev.med.59.110106.132000

[14] Wolcott R, Cutting K, Dowd S (2008) Surgical site infections: biofilms, dehiscence and delayed healing. Wounds UK 4: 108–113.

[15] Kathju S, Nistico L, Hall-Stoodley L, Post JC, Ehrlich GD, et al (2009) Chronic surgical site infection due to suture-associated polymicrobial biofilm. Surg Infect (Larchmt) 10: 457–461. 19811056 10.1089/sur.2008.062 PMC2956523

[16] Hidron AI, Low CE, Honig EG, Blumberg HM (2009) Emergence of community-acquired meticillin-resistant Staphylococcus aureus strain USA300 as a cause of necrotising community-onset pneumonia. Lancet Infect Dis 9: 384–392. 19467478 10.1016/S1473-3099(09)70133-1

[17] Engemann JJ, Carmeli Y, Cosgrove SE, Fowler VG, Bronstein MZ, et al (2003) Adverse clinical and economic outcomes attributable to methicillin resistance among patients with Staphylococcus aureus surgical site infection. Clin Infect Dis 36: 592–598. 12594640 10.1086/367653

[18] McCann MT, Gilmore BF, Gorman SP (2008) Staphylococcus epidermidis device-related infections: pathogenesis and clinical management. J Pharm Pharmacol 60: 1551–1571. 19000360 10.1211/jpp/60.12.0001

[19] Singh K, Anderson E, Harper JG (2011) Overview and management of sternal wound infection. Semin Plast Surg 25: 25–33. 22294940 10.1055/s-0031-1275168 PMC3140234

[20] Hollenbeak CS, Murphy DM, Koenig S, Woodward RS, Dunagan WC, et al (2000) The clinical and economic impact of deep chest surgical site infections following coronary artery bypass graft surgery. Chest 118: 397–402. 10936131 10.1378/chest.118.2.397

[21] Gardlund B, Bitkover CY, Vaage J (2002) Postoperative mediastinitis in cardiac surgery - microbiology and pathogenesis. Eur J Cardiothorac Surg 21: 825–830. 12062270 10.1016/s1010-7940(02)00084-2

[22] Popov AF, Schmitto JD, Jebran AF, Bireta C, Friedrich M, et al (2011) Treatment of gram-positive deep sternal wound infections in cardiac surgery–experiences with daptomycin. J Cardiothorac Surg 6: 112. 21929771 10.1186/1749-8090-6-112 PMC3184046

[23] Matros E, Aranki SF, Bayer LR, McGurk S, Neuwalder J, et al (2010) Reduction in incidence of deep sternal wound infections: random or real? J Thorac Cardiovasc Surg 139: 680–685. 20018307 10.1016/j.jtcvs.2009.10.006

[24] Schols RM, Lauwers TM, Geskes GG, van der Hulst RR (2011) Deep sternal wound infection after open heart surgery: current treatment insights. A retrospective study of 36 cases. Eur J Plast Surg 34: 487–492. 22162911 10.1007/s00238-011-0573-2 PMC3218281

[25] Otto M (2009) Staphylococcus epidermidis–the 'accidental' pathogen. Nat Rev Microbiol 7: 555–567. 19609257 10.1038/nrmicro2182 PMC2807625

[26] Donlan RM (2001) Biofilms and device-associated infections. Emerg Infect Dis 7: 277–281. 11294723 10.3201/eid0702.010226 PMC2631701

[27] Donlan RM, Costerton JW (2002) Biofilms: survival mechanisms of clinically relevant microorganisms. Clin Microbiol Rev 15: 167–193. 11932229 10.1128/CMR.15.2.167-193.2002 PMC118068

[28] Marrie TJ, Costerton JW (1984) Scanning and transmission electron microscopy of in situ bacterial colonization of intravenous and intraarterial catheters. J Clin Microbiol 19: 687–693. 6429190 10.1128/jcm.19.5.687-693.1984 PMC271156

[29] Marrie TJ, Nelligan J, Costerton JW (1982) A scanning and transmission electron microscopic study of an infected endocardial pacemaker lead. Circulation 66: 1339–1341. 7139907 10.1161/01.cir.66.6.1339

[30] Bandyk DF, Bergamini TM, Kinney EV, Seabrook GR, Towne JB (1991) In situ replacement of vascular prostheses infected by bacterial biofilms. J Vasc Surg 13: 575–583. 2027196

[31] Kjaergard HK, Tingleff J, Abildgaard U, Pettersson G (1999) Recurrent endocarditis in silver-coated heart valve prosthesis. J Heart Valve Dis 8: 140–142. 10224571

[32] Stoodley P, Nistico L, Johnson S, Lasko LA, Baratz M, et al (2008) Direct demonstration of viable Staphylococcus aureus biofilms in an infected total joint arthroplasty. A case report. J Bone Joint Surg Am 90: 1751–1758. 18676908 10.2106/JBJS.G.00838 PMC2729478

[33] Stoodley P, Braxton EE Jr, Nistico L, Hall-Stoodley L, Johnson S, et al (2010) Direct demonstration of Staphylococcus biofilm in an external ventricular drain in a patient with a history of recurrent ventriculoperitoneal shunt failure. Pediatr Neurosurg 46: 127–132. 20664301 10.1159/000319396 PMC2939992

[34] Olsson E, Friberg O, Venizelos N, Koskela A, Kallman J, et al (2007) Coagulase-negative staphylococci isolated from sternal wound infections after cardiac surgery: attachment to and accumulation on sternal fixation stainless steel wires. APMIS 115: 142–151. 17295681 10.1111/j.1600-0463.2007.apm_559.x

[35] Parsek MR, Singh PK (2003) Bacterial biofilms: an emerging link to disease pathogenesis. Annu Rev Microbiol 57: 677–701. 14527295 10.1146/annurev.micro.57.030502.090720

[36] James GA, Swogger E, Wolcott R, Pulcini E, Secor P, et al (2008) Biofilms in chronic wounds. Wound Repair Regen 16: 37–44. 18086294 10.1111/j.1524-475X.2007.00321.x

[37] Han A, Zenilman JM, Melendez JH, Shirtliff ME, Agostinho A, et al (2011) The importance of a multifaceted approach to characterizing the microbial flora of chronic wounds. Wound Repair Regen 19: 532–541. 22092791 10.1111/j.1524-475X.2011.00720.x PMC3227014

[38] Wolcott RD, Gontcharova V, Sun Y, Zischakau A, Dowd SE (2009) Bacterial diversity in surgical site infections: not just aerobic cocci any more. J Wound Care 18: 317–323. 19862869 10.12968/jowc.2009.18.8.43630

[39] Wolcott RD, Cox SB, Dowd SE (2010) Healing and healing rates of chronic wounds in the age of molecular pathogen diagnostics. J Wound Care 19: 272–278, 280–271. 20616768

[40] Wolcott RD, Dowd SE (2008) A rapid molecular method for characterising bacterial bioburden in chronic wounds. J Wound Care 17: 513–516. 19052515 10.12968/jowc.2008.17.12.31769

[41] Panuncialman J, Falanga V (2007) The science of wound bed preparation. Clin Plast Surg 34: 621–632. 17967618 10.1016/j.cps.2007.07.003

[42] Wolcott RD, Kennedy JP, Dowd SE (2009) Regular debridement is the main tool for maintaining a healthy wound bed in most chronic wounds. J Wound Care 18: 54–56. 19418781 10.12968/jowc.2009.18.2.38743

[43] Rhoads DD, Wolcott RD, Percival SL (2008) Biofilms in wounds: management strategies. J Wound Care 17: 502–508. 18978690 10.12968/jowc.2008.17.11.31479

[44] Wolcott RD, Rumbaugh KP, James G, Schultz G, Phillips P, et al (2010) Biofilm maturity studies indicate sharp debridement opens a time- dependent therapeutic window. J Wound Care 19: 320–328. 20852503 10.12968/jowc.2010.19.8.77709

[45] Engelman R, Shahian D, Shemin R, Guy TS, Bratzler D, et al (2007) The Society of Thoracic Surgeons practice guideline series: Antibiotic prophylaxis in cardiac surgery, part II: Antibiotic choice. Ann Thorac Surg 83: 1569–1576. 17383396 10.1016/j.athoracsur.2006.09.046

[46] Raad I, Hanna H, Jiang Y, Dvorak T, Reitzel R, et al (2007) Comparative activities of daptomycin, linezolid, and tigecycline against catheter-related methicillin-resistant Staphylococcus bacteremic isolates embedded in biofilm. Antimicrob Agents Chemother 51: 1656–1660. 17353249 10.1128/AAC.00350-06 PMC1855569

[47] Wolcott RD, Rhoads DD (2008) A study of biofilm-based wound management in subjects with critical limb ischaemia. J Wound Care 17: 145–148, 150–142, 154–145. 10.12968/jowc.2008.17.4.28835 18494432

[48] Lopez-Leban F, Kiran MD, Wolcott R, Balaban N (2010) Molecular mechanisms of RIP, an effective inhibitor of chronic infections. Int J Artif Organs 33: 582–589. 20963725 10.1177/039139881003300904

[49] del Pozo JL, Patel R (2007) The challenge of treating biofilm-associated bacterial infections. Clin Pharmacol Ther 82: 204–209. 17538551 10.1038/sj.clpt.6100247

[50] Rendueles O, Ghigo JM (2012) Multi-species biofilms: how to avoid unfriendly neighbors. FEMS Microbiol Rev. 10.1111/j.1574-6976.2012.00328.x 22273363

